# Supplementary material for: A novel variant of the Listeria monocytogenes type VII secretion system EssC component is associated with an Rhs toxin
Source: Microb Genom. 2023 Jun 6;9(6):mgen001036. doi: 10.1099/mgen.0.001036 (PMC10327511; doi:10.1099/mgen.0.001036)
Supplement: Supplementary material 1 [file mgen-9-1036-s001.pdf]

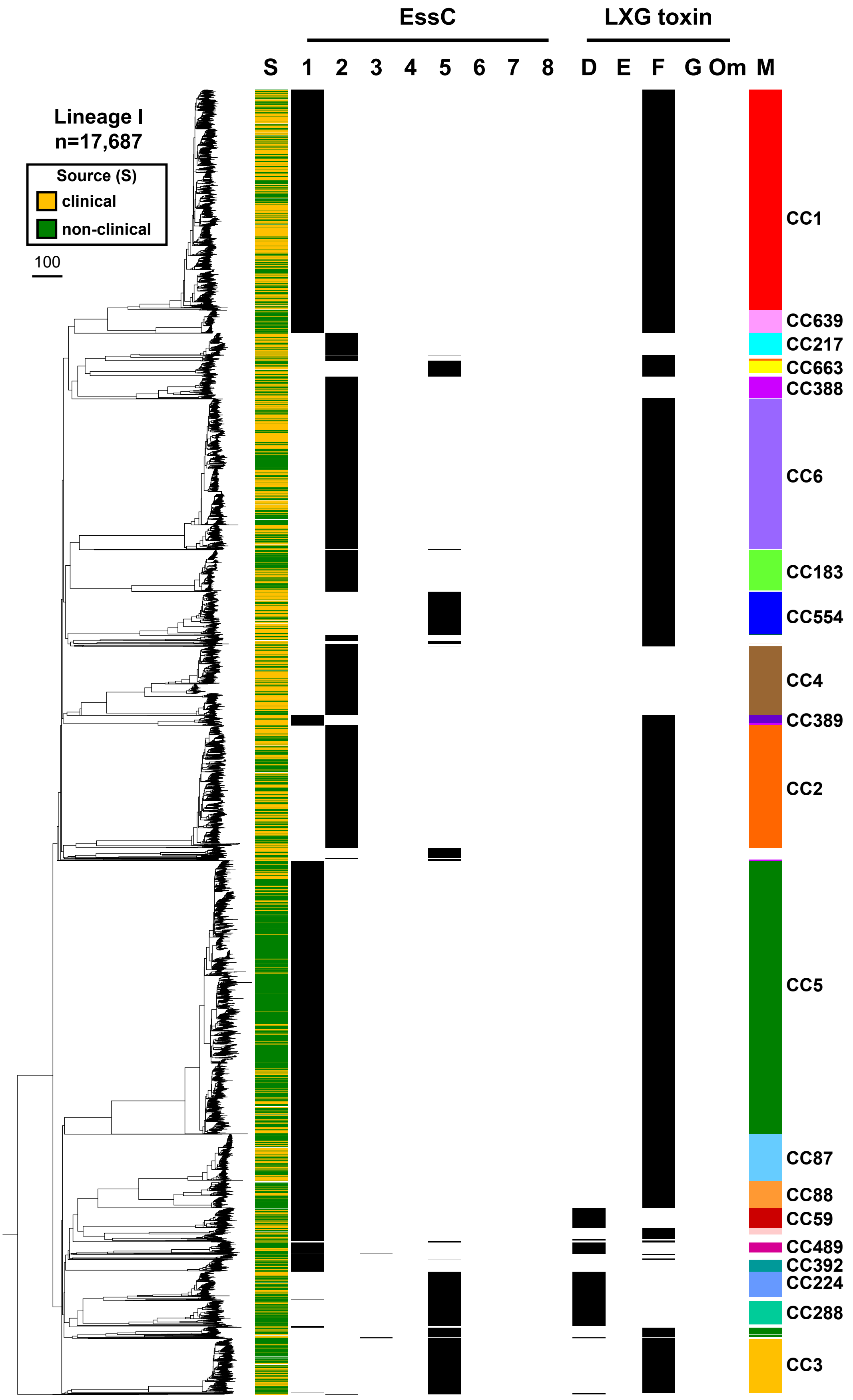
EssC
LXG toxin
S
1
2
3
4
5
6
7
8
D
E
F
G
Om
M
Lineage I
n=17,687
Source (S)
clinical
non-clinical
100
CC1
CC639
CC217
CC663
CC388
CC6
CC183
CC554
CC4
CC389
CC2
CC5
CC87
CC88
CC59
CC489
CC392
CC224
CC288
CC3

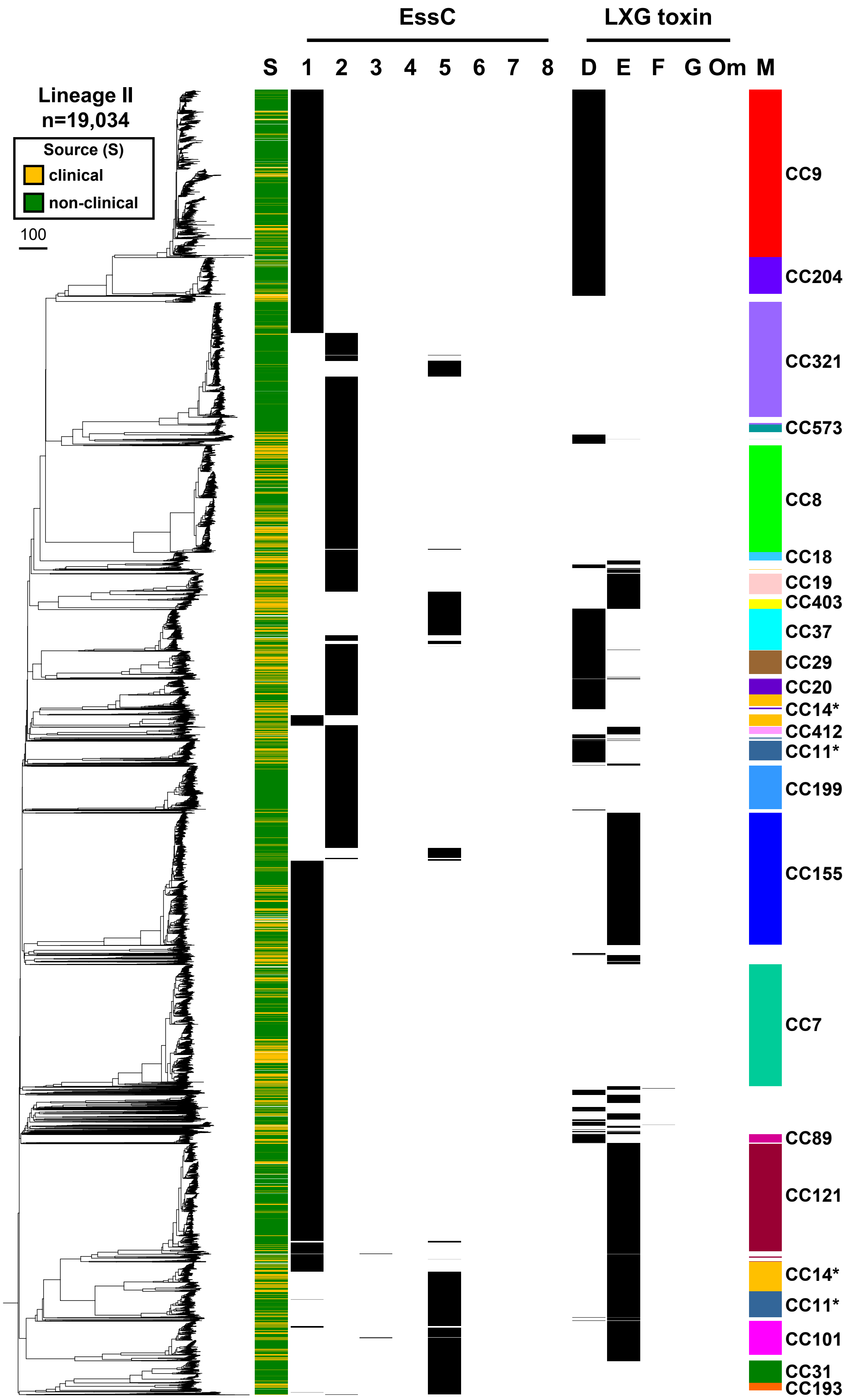
EssC
LXG toxin
S
1
2
3
4
5
6
7
8
D
E
F
G
Om
M
Lineage II
n=19,034
Source (S)
clinical
non-clinical
100
CC9
CC204
CC321
CC573
CC8
CC18
CC19
CC403
CC37
CC29
CC20
CC14*
CC412
CC11*
CC199
CC155
CC7
CC89
CC121
CC14*
CC11*
CC101
CC31
CC193

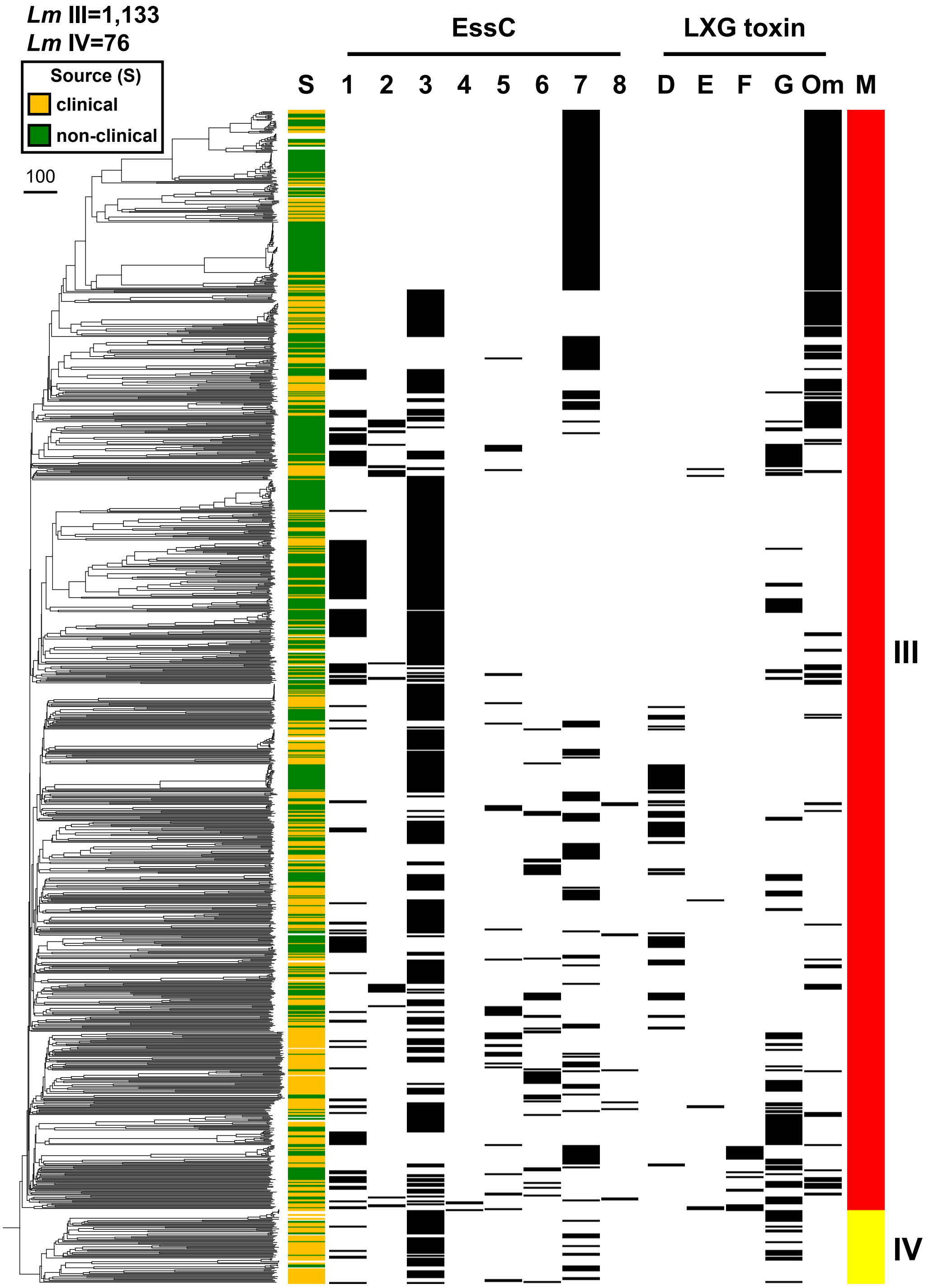

Figure S1. Phylogenetic trees of a) Lineage I b) Lineage II and c) Lineages III and IV of *L. monocytogenes* in the genome repository. The phylogenetic tree is based on core genome MLST profiles. The clonal complexes and clinical outcome are mapped onto each of the four linages, *essC* subtype and presence/identity of the LXG toxin in variable region 2 is also shown. Om – omicron.

```
EssC1    MNREALLIVSNGQQCHKNHLSPEKVVTIGNTIEHEITYPELAESIEVKYDEGSWNAGTTA  60
EssC2    MNREALLIVSNGQQCHKNHLSPEKVVTIGNTIEHEITYPELAESIEVKYDEGSWNAGTTA  60
EssC3    MNREALLIVSNGQQCHKHHLSPERVVTIGNTIEHEITYPELAESIEVKYDEGSWNAGTTA  60
EssC4    MNREALLIVSNGQQCHKNHLSPEKVVTIGNTIEHEITYPELAESIEVKYDEGSWNAGTTA  60
EssC5    MNREALLIVSNGQQCHKNHLSPEKVVTIGNTIEHEITYPELAESIEVKYDEGSWNAGTTA  60
EssC6    MNREALLIVSNGQQCHKHHLSPERVVTIGNTIEHEITYPELAESIEVKYDEGSWNAGTTA  60
EssC7    MNREALLIVSNGQQCHKHHLSPERVVTIGNTIEHEITYPELAESIEVKYDEGSWNAGTTA  60
EssC8    MNREALLIVSNGQQCHKHHLSPERVVTIGNTIEHEITYPELAESIEVIYDEGSWNAGTTA  60

EssC1    LQANQAVNVENLAFYLCQDLHTQVYDVVTNISVTFGVGIENDVTLDDTKSDFILLRDAKE  120
EssC2    LQANQAVNVENLAFYLCQDLHTQVYDVVTNISVTFGVGIENDVTLDDTKSDFILLRDAKE  120
EssC3    LQANQAVNVENLAFYLCQDLHTQVYDVVTNISVTFGVGIENDVTLDDTKSDFILLRDTKE  120
EssC4    LQANQAVNVENLAFYLCQDLHTQVYDVVTNISVTFGVGIENDVTLDDTKSDFILLRDAKE  120
EssC5    LQANQAVNVENLAFYLCQDLHTQVYDVVTNISVTFGVGIENDVTLDDTKSDFILLRDAKE  120
EssC6    LQANQAVNVENLAFYLCQDLHTQVYDVVTNISVTFGVGIENDVTLDDTKSDFILLRDTKE  120
EssC7    LQANQAVNVENLAFYLCQDLHTQVYDVVTNISVTFGVGIENDVTLDDTKSDFILLRDTKE  120
EssC8    LQANQAVNVENLAFYLCQDLHTQVYDVVTNISVTFGVGIENDVTLDDTKSDFILLRDTKE  120

EssC1    KLFKLQVLNGEVYHNFSLVTEDCVLEPGDQLYTDGVTITIGKEDISVLAVKNRVTSKLAP  180
EssC2    KLFKLQVLNGEVYHNFSLVTEDCVLEPGDQLYTDGVTITIGKEDISVLAVKNRVTSKLAP  180
EssC3    GLFKLQVLNGEIYHNFSLVTEDCTVEPGDQLYTDGVTITIGKEDISVLAVKNRVTSKLAP  180
EssC4    KLFKLQVLNGEVYHNFSLVTEDCVLEPGDQLYTDGVTITIGKEDISVLAVKNRVTSKLAP  180
EssC5    KLFKLQVLNGEVYHNFSLVTEDCVLEPGDQLYTDGVTITIGKEDISVLAVKNRVTSKLAP  180
EssC6    GLFKLQVLNGEIYHNFSLVTEDCTVEPGDQLYTDGVTITIGKEDISVLAVKNRVTSKLAP  180
EssC7    GLFKLQVLNGEIYHNFSLVTEDCTVEPGDQLYTDGVTITIGKEDISVLAVKNRVTSKLAP  180
EssC8    GLFKLQVLNGEIYHNFSLVTEDCTVEPGDQLYTDGVTITIGKEDISVLAVKNRVTSKLAP  180

EssC1    LFAADNSFGEDYPDYHRSPRIIYRAPEEKISMAKPSSKPSKPTDGLIKIILPPLIMVAIT  240
EssC2    LFAADNSFGEDYPDYHRSPRIIYRAPEEKISMAKPSSKPSKPTDGLIKIILPPLIMVAIT  240
EssC3    LFAADNSFGEDYPDYHRSPRIIYRAPEEKISMAKPSSKPSKPTDGLIKIILPPLIMVAIT  240
EssC4    LFAADNSFGEDYPDYHRSPRIIYRAPEEKISMAKPSSKPSKPTDGLIKIILPPLIMVAIT  240
EssC5    LFAADNSFGEDYPDYHRSPRIIYRAPEEKISMAKPSSKPSKPTDGLIKIILPPLIMVAIT  240
EssC6    LFAADNSFGEDYPDYHRSPRIIYRAPEEKISMAKPSSKPSKPTDGLIKIILPPLIMVAIT  240
EssC7    LFAADNSFGEDYPDYHRSPRIIYRAPEEKISMAKPSSKPSKPTDGLIKIILPPLIMVAIT  240
EssC8    LFAADNSFGEDYPDYHRSPRIIYRAPEEKISMAKPSSKPSKPTDGLIKIILPPLIMVAIT  240

EssC1    VMISIFQPRGLYIIMTIAMSAVTITMAVLNYIKSRKKYKIDSKQRVESYDLYLKRKTKEL  300
EssC2    VMISIFQPRGLYIIMTIAMSAVTITMAVLNYIKSRKKYKIDSKQRVESYDLYLKRKTKEL  300
EssC3    VMISIFQPRGLYIIMTIAMSAVTITMAILNYIKSRKKYKIDSKQRVESYDLYLKRKTKEL  300
EssC4    VMISIFQPRGLYIIMTIAMSAVTITMAILNYIKSRKKYKIDSKQRVESYDLYLKRKTKEL  300
EssC5    VMISIFQPRGLYIIMTIAMSAVTITMAILNYIKSRKKYKIDSKQRVESYDLYLKRKTKEL  300
EssC6    VMISIFQPRGLYIIMTIAMSAVTITMAILNYIKSRKKYKIDSKQRVESYDLYLKRKTKEL  300
EssC7    VMISIFQPRGLYIIMTIAMSAVTITMAILNYIKSRKKYKIDSKQRVESYDLYLKRKTKEL  300
EssC8    VMISIFQPRGLYIIMTIAMSAVTITMAILNYIKSRKKYKIDSKQRVESYDLYLKRKTKEL  300

EssC1    HETSEKQRHALTYHYPDVTELEKMALRVDSRIYEKTMFHHDFLTFRVGRGDEASSFSVEF  360
EssC2    HETSEKQRHALTYHYPDVTELEKMALRVDSRIYEKTMFHHDFLTFRVGRGDETSSFSVEF  360
EssC3    HETSEKQRHALTYHYPDVTELEKMALRVDSRIYEKTMFHHDFLTFRVGRGDETSSFSVEF  360
EssC4    HETSEKQRHALTYHYPDVTELEKMALRVDSRIYEKTMFHHDFLTFRVGRGDEASSFSVEF  360
EssC5    HETSEKQRHALTYHYPDVTELEKMALRVDSRIYEKTMFHHDFLTFRVGRGDETSSFSVEF  360
EssC6    HETSEKQRHALTYHYPDVTELEKMALRVDSRIYEKTMFQHDFLTFRVGRGDEASSFSVEF  360
EssC7    HETSEKQRHALTYHYPDVTELEKMALRVDSRIYEKTMFHHDFLTFRVGRGDEASSFSVEF  360
EssC8    HETSEKQRHALTYHYPDVTELEKMALRVDSRIYEKTMFHHDFLTFRVGRGDEASSFSVEF  360

EssC1    QQEEFSQEKDELVEEAVKIKGQYLSINEVPVATDLMHGPVGYIGPRRLVLEQLQMLVMQT  420
EssC2    QQEEFSQEKDELVEEAVKIKGQYLSINEVPVATDLMHGPVGYIGPRRLVLEQLQMLVMQT  420
EssC3    QQEEFSQEKDELVEEAVKIKGQYLSINEVPVATDLMHGPVGYIGPRRLVLEQLQMLVMQT  420
EssC4    QQEEFSQEKDELVEEAVKIKGQYLSINEVPVATDLMHGPVGYIGPRRLVLEQLQMLVMQT  420
EssC5    QQEEFSQEKDELVEEAVKIKGQYLSINEVPVATDLMHGPVGYIGPRRLVLEQLQMLVMQT  420
EssC6    QQEEFSQEKDELVEEAVKIKGQYLSINEVPVATDLMHGPVGYIGPRRLVLEQLQMLVMQT  420
EssC7    QQEEFSQEKDELVEEAVKIKGQYLSINEVPVATDLMHGPVGYIGPRRLVLEQLQMLVMQT  420
EssC8    QQEEFSQEKDELVEEAVKIKGQYLSINEVPVATDLMHGPVGYIGPRRLVLEQLQMLVMQT  420
```

```
EssC1  SLFHSYYDLQFITIFPEEEKADWDWMRWLPHANMRDVNVRGFVYHERSRDQVLNSLYQIL 480
EssC2  SLFHSYYDLQFITIFPEEEKADWDWMRWLPHANMRDVNVRGFVYHERSRDQVLNSLYQIL 480
EssC3  SLFHSYYDLQFITIFPEEEKADWDWMRWLPHANMRDVNVRGFVYHERSRDQVLNSLYQIL 480
EssC4  SLFHSYYDLQFITIFPEEEKADWDWMRWLPHANMRDVNVRGFVYHERSRDQVLNSLYQIL 480
EssC5  SLFHSYYDLQFITIFPEEEKVDWDWMRWLPHANMRDVNVRGFVYHERSRDQVLNSLYQIL 480
EssC6  SLFHSYYDLQFITIFPEEEKADWDWMRWLPHANMRDVNVRGFVYHERSRDQVLNSLYQIL 480
EssC7  SLFHSYYDLQFITIFPEEEKADWDWMRWLPHANMRDVNVRGFVYHERSRDQVLNSLYQIL 480
EssC8  SLFHSYYDLQFITIFPEEEKPDWDWMRWLPHANMRDVNVRGFVYHERSRDQVLNSLYQIL 480

EssC1  KERKQALTEQASKQEKLYFTPHYVVLITDEKLVLDHTVMEFFNEDPSELGVSLVFVQDVM 540
EssC2  KERKQALTEQASKQEKLYFTPHYVVLITDEKLVLDHTVMEFFNEDPSELGVSLVFVQDVM 540
EssC3  KERKQALTEQASKQEKLYFTPHYVVLITDEKLVLDHTVMEFFNEDPSELGVSLVFVQDVM 540
EssC4  KERKQALTEQASKQEKLYFTPHYVVLITDEKLVLDHTVMEFFNEDPSELGVSLVFVQDVM 540
EssC5  KERKQALTEQASKQEKLYFTPHYVVLITDEKLVLDHTVMEFFNEDPSELGVSLVFVQDVM 540
EssC6  KERKQALTEQASKQEKLYFTPHYVVLITDEKLVLDHTVMEFFNEDPSELGVSLVFVQDVM 540
EssC7  KERKQALTEQASKQEKLYFTPHYVVLITDEKLVLDHTVMEFFNEDPSELGVSLVFVQDVM 540
EssC8  KERKQALTEQASKQEKLYFTPHYVVLITDEKLVLDHTVMEFFNEDPSELGVSLVFVQDVM 540

EssC1  ESLPEHVKTVVDIRDAKSGNIILEQGDLVNRAFVPDHLPADFDKEVISRALAPLNHLQNL 600
EssC2  ESLPEHVKTVVDIRDAKSGNIILEQGDLVNRAFVPDHLPADFDKEVISRALAPLNHLQNL 600
EssC3  ESLPEHVKTVVDIRDAKSGNIILEQGDLVNRAFVPDHLPADFDKEVISRALAPLNHLQNL 600
EssC4  ESLPEHVKTVVDIRDAKSGNIILEQGDLVNRAFVPDHLPADFDKEVISRALAPLNHLQNL 600
EssC5  ESLPEHVKTVVDIRDAKSGNIILEQGDLVNRAFVPDHLPADFDKEVISRALAPLNHLQNL 600
EssC6  ESLPEHVKTVVDIRDAKSGNIILEQGDLVNRAFVPDHLPADFDKEVISRALAPLNHLQNL 600
EssC7  ESLPEHVKTVVDIRDAKSGNIILEQGDLVNRAFVPDHLPADFDKEVISRALAPLNHLQNL 600
EssC8  ESLPEHVKTVVDIRDAKSGNIILEQGDLVNRAFVPDHLPADFDKEVISRALAPLNHLQNL 600

EssC1  KNSIPESVTFLEMYGVERVEELNIAGRWAKNETYKSLAVPLGLRGKDDIVQLNLHEKAHG 660
EssC2  KNSIPESVTFLEMYGVERVEELNIAGRWAKNETYKSLAVPLGLRGKDDIVQLNLHEKAHG 660
EssC3  KNSIPESVTFLEMYGVERVEELNIAGRWAKNETYKSLAVPLGLRGKDDIVQLNLHEKAHG 660
EssC4  KNSIPESVTFLEMYGVERVEELNIAGRWAKNETYKSLAVPLGLRGKDDIVQLNLHEKAHG 660
EssC5  KNSIPESVTFLEMYGVERVEELNIAGRWAKNETYKSLAVPLGLRGKDDIVQLNLHEKAHG 660
EssC6  KNSIPESVTFLEMYGVERVEELNIAGRWAKNETYKSLAVPLGLRGKDDIVQLNLHEKAHG 660
EssC7  KNSIPESVTFLEMYGVERVEELNIAGRWAKNETYKSLAVPLGLRGKDDIVQLNLHEKAHG 660
EssC8  KNSIPESVTFLEMYGVERVEELNIAGRWAKNETYKSLAVPLGLRGKDDIVQLNLHEKAHG 660

EssC1  PHGLVAGTTGSGKSEIIQSYIISLGVNFHPYEVAFLLIDYKGGGMANLFKNMPHLLGTIT 720
EssC2  PHGLVAGTTGSGKSEIIQSYIISLGVNFHPYEVAFLLIDYKGGGMANLFKNMPHLLGTIT 720
EssC3  PHGLVAGTTGSGKSEIIQSYIISLGVNFHPYEVAFLLIDYKGGGMANLFKNMPHLLGTIT 720
EssC4  PHGLVAGTTGSGKSEIIQSYIISLGVNFHPYEVAFLLIDYKGGGMANLFKNMPHLLGTIT 720
EssC5  PHGLVAGTTGSGKSEIIQSYIISLGVNFHPYEVAFLLIDYKGGGMANLFKNMPHLLGTIT 720
EssC6  PHGLVAGTTGSGKSEIIQSYIISLGVNFHPYEVAFLLIDYKGGGMANLFKNMPHLLGTIT 720
EssC7  PHGLVAGTTGSGKSEIIQSYIISLGVNFHPYEVAFLLIDYKGGGMANLFKNMPHLLGTIT 720
EssC8  PHGLVAGTTGSGKSEIIQSYIISLGVNFHPYEVAFLLIDYKGGGMANLFKNMPHLLGTIT 720

EssC1  NLDGAQSMRALASIKAELQKRQRLFGEHDVNHINQYQKLYKQGKATEPMPHLFLISDEFA 780
EssC2  NLDGAQSMRALASIKAELQKRQRLFGEHDVNHINQYQKLYKQGKATEPMPHLFLISDEFA 780
EssC3  NLDGAQSMRALASIKAELQKRQRLFGEHDVNHINQYQKLYKQGKATEPMPHLFLISDEFA 780
EssC4  NLDGAQSMRALASIKAELQKRQRLFGEHDVNHINQYQKLYKQGKATEPMPHLFLISDEFA 780
EssC5  NLDGAQSMRALASIKAELQKRQRLFGEHDVNHINQYQKLYKQGKATEPMPHLFLISDEFA 780
EssC6  NLDGAQSMRALASIKAELQKRQRLFGEHDVNHINQYQKLYKQGKATEPMPHLFLISDEFA 780
EssC7  NLDGAQSMRALASIKAELQKRQRLFGEHDVNHINQYQKLYKQGKATEPMPHLFLISDEFA 780
EssC8  NLDGAQSMRALASIKAELQKRQRLFGEHDVNHINQYQKLYKQGKATEPMPHLFLISDEFA 780
                                                         *
EssC1  ELKSEQPEFMKELVSTARIGRSLGIHLILATQKPSGVVDDQIWSNSKFKLALKVQNASDS 840
EssC2  ELKSEQPEFMKELVSTARIGRSLGIHLILATQKPSGVVDDQIWSNSKFKLALKVQNASDS 840
EssC3  ELKSEQPEFMKELVSTARIGRSLGIHLILATQKPSGVVDDQIWSNSKFKLALKVQNASDS 840
EssC4  ELKSEQPEFMKELVSTARIGRSLGIHLILATQKPSGVVDDQIWSNSKFKLALKVQNASDS 840
EssC5  ELKSEQPEFMKELVSTARIGRSLGIHLILATQKPSGVVDDQIWSNSKFKLALKVQNASDS 840
EssC6  ELKSEQPEFMKELVSTARIGRSLGIHLILATQKPSGVVDDQIWSNSKFKLALKVQNASDS 840
EssC7  ELKSEQPEFMKELVSTARIGRSLGIHLILATQKPSGVVDDQIWSNSKFKLALKVQNASDS 840
EssC8  ELKSEQPEFMKELVSTARIGRSLGIHLILATQKPSGVVDDQIWSNSKFKLALKVQNASDS 840
```

```
EssC1   NEILKTPDAAEITLPGRSYLQVGNNEIYELFQSAWSGADYVPDKESTDYIDTTIYAINDL  900
EssC2   NEILKTPDAAEITLPGRSYLQVGNNEIYELFQSAWSGADYVPDKESTDYIDTTIYAINDL  900
EssC3   NEILKTPDAAEITLPGRSYLQVGNNEIYELFQSAWSGADYVPDKESTDYIDTTIYAINDL  900
EssC4   NEILKTPDAAEITLPGRSYLQVGNNEIYELFQSAWSGADYVPDKESTDYIDTTIYAINDL  900
EssC5   NEILKTPDAAEITLPGRSYLQVGNNEIYELFQSAWSGADYVPDKESTDYIDTTIYAINDL  900
EssC6   NEILKTPDAAEITLPGRSYLQVGNNEIYELFQSAWSGADYVPDKESTDYIDTTIYAINDL  900
EssC7   NEILKTPDAAEITLPGRSYLQVGNNEIYELFQSAWSGADYVPDKESTDYIDTTIYAINDL  900
EssC8   NEILKTPDAAEITLPGRSYLQVGNNEIYELFQSAWSGADYVPDKESTDYIDTTIYAINDL  900

EssC1   GQYDILTEDLSGLDKKDDLTKLPSELDAVIDHIHEYTEASGIEALPRPWLPPLEEQIFLP  960
EssC2   GQYDILTEDLSGLDKKDDLTKLPSELDAVIDHIHEYTEASGIEALPRPWLPPLPERIFAQ  960
EssC3   GQYDILTEDLSGLDKKDDLTKLPSELDAVIDHIHEYTEASGIEALPRPWLPPLEERISLE  960
EssC4   GQYDILTEDLSGLDKKDDLTKLPSELDAVIDHIHEYTEASGIEALPRPWLPPLPERIFAQ  960
EssC5   GQYDILTEDLSGLDKKDDLTKLPSELDAVIDHIHAYTEASGIEALPRPWLPPLPERIFAQ  960
EssC6   GQYDILTEDLSGLDKKDDLTKLPSELDAVIDHIHEYTEASGIEALPRPWLPPLPERIFAQ  960
EssC7   GQYDILTEDLSGLDKKDDLTKLPSELDAVIDHIHEYTEASGIEALPRPWLPPLPERIFAQ  960
EssC8   GQYDILTEDLSGLDKKDDLTKLPSELDAVIDHIHEYTEASGIEALPRPWLPPLEEQIFLP  960

EssC1   ELHQVITDELWSGEKQPLQATIGFLDIPQMQAQEPLTIDLAKDGHLAVFSSPGYGKSTFL  1020
EssC2   DLHPVNFEEAWKEPKKPLQATIGLLDQPELQAQVPLTLDLTKDGHVAVFSSPGFGKSTFL  1020
EssC3   SISTVDFEANWQKDEKDLELTLGVLDQPQLQAQNVLHWNLEKNGHMAVFSSPGFGKSTFM  1020
EssC4   DLHPVNFEEAWKEPKKPLQATIGLLDQPELQAQVPLTLDLTKDGHVAVFSSPGFGKSTFL  1020
EssC5   DLHPVNFEEAWKEPKKPLQATIGLLDQPELQAQVPLTLDLTKDGHVAVFSSPGFGKSTFL  1020
EssC6   DLHPVNFEEAWKEPKKPLQATIGLLDQPELQAQVPLTLDLTKDGHVAVFSSPGFGKSTFL  1020
EssC7   DLHPVNFEEAWKEPKKPLQATIGLLDQPELQAQVPLTLDLTKDGHVAVFSSPGFGKSTFL  1020
EssC8   ELHQVITDELWSGEKQPLQATIGFLDIPQMQAQEPLTIDLAKDGHLAVFSSPGYGKSTFL  1020

EssC1   QTITMDLARQHNPERLHIYLLDLGTNGLLPLKKLPHVADTIMVDEEIKIGKLIRRLTLEL  1080
EssC2   QSLVMDLARQHNPEQLHVYLLDFGTNGLLPLVDLPHIADTMMVDEVEKIQKFLRICLNEI  1080
EssC3   QTAIFDLARKNTPEFFHAYLLDFGTNGLLSLKGLPHVADTFSIDETEKTLKLVRLLSREI  1080
EssC4   QSLVMDLARQHNPEQLHVYLLDFGTNGLLPLIDLPHVADTMMVDEVEKVQKFVRRISEVM  1080
EssC5   QSLVMDLARQHNPEQLHVYLLDFGTNGLLPLIDLPHVADTIMIDEIEKARKFVKIVIREI  1080
EssC6   QSLVMDLARQHNPEQLHVYLLDFGTNGLLPLAKLPHTVDLLNIDNEEKLRKFTNRMNELI  1080
EssC7   QSLVMDLARQHNPEQLHVYLLDFGTNGLLSMKDVPHVADLMRLDEEEKITKLLKRVQNEI  1080
EssC8   QTITMDLARQHNPERLHIYLLDLGTNGLLPLKKLPHVADTIMVDEEIKIGKLIRRLYQEL  1080

EssC1   KERKQKLSKYGVASISMYEKASKEEVPAILLVIDAFDSVGEAP-YKDVFEKLIAQIAREG  1139
EssC2   KTRKKLLSQYRVANIEQYERASGKELPNIIIVLDNYDAVKDAG-LGDDFEKIITQITREG  1139
EssC3   KERKQLLSKFSVASLKMYEEISGDKKPIILLAIDNYDAIREVDEFVANLEPTIVQIAREG  1140
EssC4   KYRKKLLSKYRVANIEQYEKASKEEIPNIVIVLDNFDAVREAG-FGENFDKIMGQVSREG  1139
EssC5   KVRKKLLSEYRVANIEQYSQASGKNVANILVCLDNYDALREAG-FGDEFDKTMIQMAREG  1139
EssC6   SDRKKLLSKNAVANLSQFEEITKEVLPEVLVLIDNYDAIRETD-FSFGFESTLTQIAREG  1139
EssC7   ETRKKLLSEYSVASLEQYEKASQKQLPHILITLDGYDVVRDSD-LPPEFEKMLIQITREG  1139
EssC8   KIRKQKLSQYGVANISMYERASGEEIPSILLVIDAYDSISQAD-YKDSLEKIVAQIAREG  1139

EssC1   ASVGIHLVMSAVRQNAIRVQMLASIKHQIPLFMIEPGEARSIVGKTDLTIEELPGRGLVK  1199
EssC2   ASIGMFMIISASRHMSLRTQMATNIKQMIALYLIDKNDISSTVGRTDTPLEEYPGRALVK  1199
EssC3   ASLGIHLMITANNQNAMRLQLLSNIKTQIALHLNEKNEVSSIVGRSDYTIEELPGRGLIK  1200
EssC4   SSVGVFLATSASKYTSIKMQIVANIKLMVSLFIIDISDTRAIVGRTDLSVEELAGRGLVN  1199
EssC5   AALGIYLVTSASKQSSIRMQVMSSIKLQIALYLIDKSEVTSIVGRTDLILEELYGRGMVK  1199
EssC6   NSVGIHLVMSATRQNSMRQNLLANLKLQLALYMIEGNEVKSIVGQTKLTVAEFAGRGLVK  1199
EssC7   AAIGIHLALSAIRGAAMKPQMLMNFKLVVSLFNIDLSESRALIGRTDLTIEEIAGRGMVK  1199
EssC8   AGVGIHLVTSAVRQSAIRVQVLANIKNQISLYLIDQNEAKSIVGRTDLKIDEIPGRGLVK  1199

EssC1   LEEPTVFQTALPVCADGTLEIIEKIQAESEAMSSEWNGSRPAPIPMVPEVINMLEYLENK  1259
EssC2   LETVTTFQTTLPSQGEETIQQIEGIRNEAKLMREGWQGELPESVPMIPEKIKLADFCEWM  1259
EssC3   IEEPTLFQMALPNNGAEAIEIIKNNQDEAEKMTEIWTGQKPQCIPMVPETLGFTHFTDHM  1260
EssC4   QDGVSVMQSILPADGEDVLEQISNLQSEAKSMRCAWKGHLPEDIPMMPDELTFTNFITKE  1259
EssC5   VGSQAIFQTTLPTKGREIVEQINNLTEEIAVMKESWNGECPDSIPVMPEVLSISDFVGRK  1259
EssC6   LEDPTLFQASFPTRSNSVTEHLECLDKEILQMSKHGIYKVVPKIPMLPDKIGLDELISKK  1259
EssC7   LEHPTIFQVNTPTEGDDILETIANIKIEAKKMEESWNGERPEAIPVVPEKMEYNAFATMP  1259
EssC8   LEDPTSFQAALPVAGSDTLEIIDKVQKECQVMRDLWKGILPISIPMVPEEFSIKQFLSMD  1259
```

```
EssC1          QVQKSLSE-GKTPIAVDFEDVLPVNLDVQTDGNTLVLTDNADILERTM----VSLTEL-I 1313
EssC2          ETKEVVAN-GNIPIGVDFEYVKPVGIDYKQTGVLIIADATGRNLVSIN----KNIANS-L 1313
EssC3          ETKKMLELKSILPIGLEYEYASPVGVSLEQHNLAIIMPTVELVQQII-----TNSSHL-L 1314
EssC4          SVK-NINN-NEIAVGLDFEVVEGVRWDLQKLDNILILGGSQAGKTTMQHTLLKGIERANL 1317
EssC5          TTKDNIQS-EILPLGLEYENIEAVGLDFKLKQNLLIVSDSNIKLNVVQ----QSIVKSGQ 1314
EssC6          SVQQSLAN-HLFVIGLDEEAVESFAVNKINQLPLLVTGENGFDISNAI----KQFIDLES 1314
EssC7          YVQKLMES-NLVPIGFDMETALPVGLDLNKQRIWLITGSETDLNENTFHAMIKGIQKI-- 1316
EssC8          KTKEYLDVYGELPIGIDFEYAEAVRVALYKQNLVVVTTTSQELIEKL-----NSLINI-L 1313

EssC1          GQNMEVDIALYDNSSNRFQQYRNN--VNIYAGEETAMNSASAQMVSVLEAREDGWKEIQR 1371
EssC2          VQNPNISLLISDTANFDLVEMSTK--ANLYSNNTEDLKQNMKKVLSIYQSRTEKADELRK 1371
EssC3          VKKELRELVVFDTPSMELMKLKEL--PLTYITNSERIEGKIDILYGAFKTAEQAYLEELQ 1372
EssC4          MKRDTNIIHIFDTNRGKLKEYKEKEIVANYIVDKDAYGEFFNGLSELLEYRFNELLRIRD 1377
EssC5          SVKSSYQLLILDKISQPLKDVSVW--ANGYVSKLEEYEKFTSMMEEEFATRKD------- 1365
EssC6          KDFESKEIIVIDNMKYDFSKYQND--IYIYGNNKTFMETIIQNSMNEYEERRKVVTRLRE 1372
EssC7          ----GQKLHLVDLSNYNLANLNSE--NTNFISKPETFQEVIKEVYAENNVREEAVRKCSP 1370
EssC8          TKKSDVETTVIDQKAMELISINDN--VSDYITDIESVETFIEDVYDDYREREAAFQEEIQ 1371

EssC1          ASNGEITLKMYVEELRPMYVLLTDSIYAAEQMSLEARKNIVRLIENGPKLGIYFVTGSQT 1431
EssC2          -AGDNLGVTAMIQGLKDIVVFVGDYDILLKALDEEDQKTLIDMINNGKTYGCYLILSGSI 1430
EssC3          -SNNTITKEEYFKNVPPIVVVTYSTVQLYNQLSSNAQKQLLEMLKSDGKMGVEFIIGNDL 1431
EssC4          -MDGEKAGEEFIQGEQKHFIFIQNIGDFGNGLPTEVVKKLVYILDNNSKLGIHFIISGTS 1436
EssC5          -----LDSKALLKDTEGWIVLISDLAEFIQH-SSITIERMKEFVENGPNLNIFFIISGMQ 1419
EssC6          ENRTRIEIEEQLANMPEILLFITDLSSVIESVEAKYLDMLEKMLGDAAVYKVFFTIGASL 1432
EssC7          -----PELNAYYQTIETINIAV-DTLEVFSKVDSETQRMIQEMMTSRIQTNINIIIQVDI 1424
EssC8          QSNGQTSPIDFYKKYKAKCIIFNGFIQIQNDLSTRTQGQLLELLKQEGKTGIWFVIGNEL 1431

EssC1          GILYRARDEISGELR-KQKTGILLGRISDQNILSLINNIYKEALLAPYEAYYIKQGQYEK 1490
EssC2          QAF-KGYGDLNKAVQ-AQRNYIFFDRLSNSSPFSFSFNIYREQELLPEEGYVIAGQEWQK 1488
EssC3          GSMAKEYSPIGDAIR-NSKQVLLGARFADQAIYSPTIRIVQEKPLAPQELYLLLEGNAEK 1490
EssC4          NNFGQNYSDFTNRVK-QINSGIIIAGYNEQSIVRMDNVNMYSPKLGAGDAYFVDNGRATR 1495
EssC5          TSIEYGLNPIEKFVKTTIRTGLVAMKNADQGIFK-GNYISNEQPLEKFETYFYVDSTRQK 1478
EssC6          GSLS-GYDSLSKTVK-AMKNVLLFTDVKQQSVINVVSKSGILKPLKPFEAYYIDNKIYSK 1490
EssC7          SDIGRDGSSVGRELR-NVTNLILSMKIKNQTTFAQDIRSYDEPDLPPRTSYYINGKLASK 1483
EssC8          GIIGKAYGDLANKVK-ESKLVILAMRVTDQTVFSTINRIVQEQQLRKGEVYLIANNQPQK 1490

EssC1          IKLIAPNQ-- 1498
EssC2          IKIAKYEEEV 1498
EssC3          LKIPKG---- 1496
EssC4          IRMPKH---- 1501
EssC5          IKLPN----- 1483
EssC6          VKVAYVKEEE 1500
EssC7          FRVLSKS--- 1490
EssC8          VKIGV----- 1495
```

	EssC1	EssC2	EssC3	EssC4	EssC5	EssC6	EssC7	EssC8
EssC1	100.00							
EssC2	77.87	100.00						
EssC3	77.24	76.76	100.00					
EssC4	77.44	80.64	75.55	100.00				
EssC5	78.12	81.49	77.50	81.16	100.00			
EssC6	76.89	77.56	76.62	77.64	78.06	100.00		
EssC7	77.52	79.73	77.22	79.03	79.86	78.72	100.00	
EssC8	82.80	77.88	79.72	77.61	78.18	77.36	78.22	100.00

Figure S2. Sequence alignment of the eight EssC variants found in *L. monocytogenes*. The shaded lines above the sequence indicate the approximate domain boundaries of EssC; red – FHA domains; grey – transmembrane domains; yellow – D0; blue – D1; orange – D2; green D3. The asterisk below His771 indicates the position at which the truncated EssC proteins encoded by the *essC* pseudogenes start to align with the full-length protein. The boxes indicate the percentage identity between each variant.

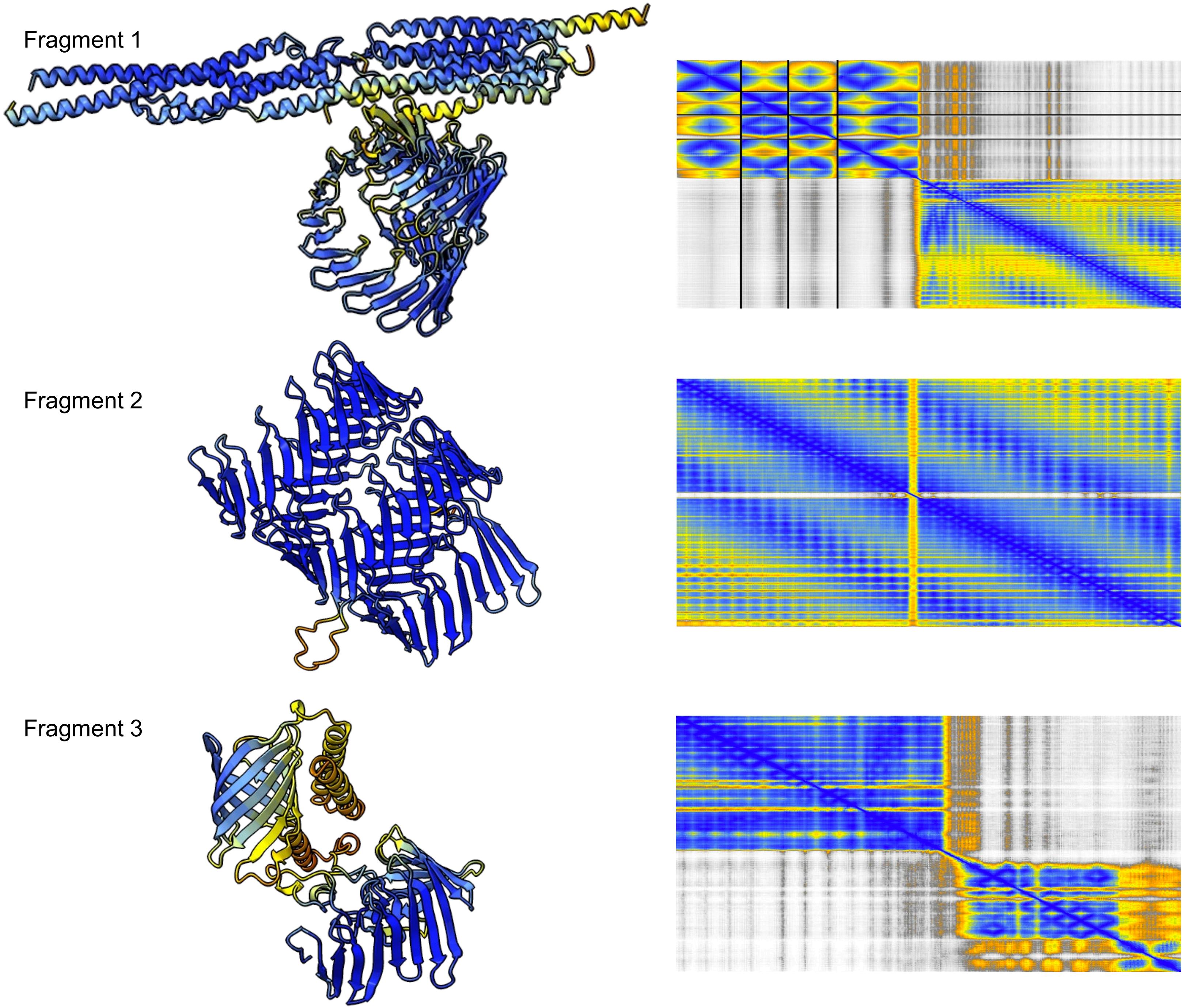

Figure S3. Individual AlphaFold models used to generate the composite LhrA model in complex with LxpABC in Fig. 4. Each model is coloured with its corresponding predicted aligned error generated by AlphaFold.

A

```
Toxin_D         MSRIDIGEIQAFLYQLRAANEPGRKTIQSIKAAVTKYVGDNSLKGKAVDASKNYYQMTYF 60
Toxin_E         MSRIDIGEIQAFLYQLRAANEPGRKTIQSIKAAVTKYVGDNSLKGKAVDASKNYYQMTYF 60
Toxin_F         MSRIDIGEIQAFLYQLRAANEPGRKTIQSIKAAVTKYVGDNSLKGKAVDASKNYYQMTYF 60
Toxin_G         MSRIDIGEIQAFLYQLRAANESGRKTIQSIKTAVTKYVGDNSLKGKAVDTSKNYYQMTYF 60
Toxin_Omicron   MSRIDIGEIQVFLYQLRAANESGRKTIQSIKTAVTKYVGDNSLKGKAVDASKTYYQMTYF 60

Toxin_D         PLCDAIIEAMDESEERLGQYIQDFHAEVDSSPDAKIDADGLYELGKMIDRIESKKEALAQ 120
Toxin_E         PLCDAIIEAMDESEERLGQYIQDFHAEVDSSPDAKIDADGLYELGKMIDRIESKKEALAQ 120
Toxin_F         PLCDAIIEAMDESEERLGQYIQDFHAEVDSSPDAKIDADGLYELGKMIDRIESKKEALAQ 120
Toxin_G         PLCDAIIEAMDESEERLGQYIQDFHAEVDSSPDAKIDADGLYELGKMIDRIESKKEALAQ 120
Toxin_Omicron   PLCDAIIEAMDESEERLGQYIQDFHAEVDSSPDAKIDADGLYELGKMIDRIESKKETLAQ 120

Toxin_D         RMNSGTEGQMQNYRSQLAIAYKQENILEKYLSFEQSHANFFDHLIDLVQAVQQTIRELQS 180
Toxin_E         RMNSGTEGQMQNYRSQLAIAYKQENILEKYLSFEQSHANFFDHLIDLVQAVQQTIRELQS 180
Toxin_F         RMNSGTEGQMQNYRSQLAIAYKQENILEKYLSFEQSHASFFDHLIDLVQAVQQTIRELQS 180
Toxin_G         RMNSGTEGQMQNYRSQLAIAYKQENILEKYLSFEQSHASFFDHLIDLVQAVQQTIRELQS 180
Toxin_Omicron   RMNSGTEGQMQNYRSQLAIAYKQENILEKYLSFEQSHASFFDHLIDLVQAVQQTIRELQS 180

Toxin_D         NIQFNSQTGTYDLSKLNSATVSRMQQALNKSRGIKEDIIKELRDYTVLAVVYLDSNGKEQ 240
Toxin_E         NIQFNSQTGTYDLSKLNSATVSRMQQALNKSRGIKEDIIKELRDYTVLAVVYLDSNGKEQ 240
Toxin_F         NIQFNSQTGTYDLSKLNHATVSHMQQALNKARGIKEDIIKELQDYTVLAVVYLDSNGKEQ 240
Toxin_G         NIQFNSQTGTYDLSKLNSATVSRMQQALNKSRGIKEDIIKELRDYTVLAVVYLDSNGKEQ 240
Toxin_Omicron   NIQFNSQTGTYDLSKLNSATVSRMQQALNKSRGIKEDIIKELRDYTVLAVVYLDSNGKEQ 240

Toxin_D         VMWLLERDGVGVENAELKAYLTENGKYLNPEDYTIITNEELNKKINQSWRDGVYYLNGNK 300
Toxin_E         VMWLLERDGLGVENAELKAYLEKNGKYLNPEDYSIITNEDLNKKINKAWRDGVYYLNGNK 300
Toxin_F         VMWLLERDGLGVENAELKAYLEKNGKYLNPEDYSIITNEDLNKKINKAWRDGVYYLNGNK 300
Toxin_G         VMWLLERDGVGVENAELKAYLTENGKYLNPEDYTIITNEELNKKINQSWRDGVYYLNGNK 300
Toxin_Omicron   VMWLLERDGVGVENAELKAYLTENGKYLNPEDYTIITNEELNKKINQSWRDGVYYLNGNK 300

Toxin_D         YDGLTGGVLSTSAYVEAGKGYIDKSGFADVVLGLGLSTAAIRGSLVYKKVN--------- 351
Toxin_E         YDGLTGGVLSTSAYVEAGKGYIDKSGLADVVLGLGLSTAAIRNATVIKSKS--------- 351
Toxin_F         YDGLTGGILSTSAYVEAGKGFIDKSGLADVVLGLGLSTAAIRGSVTFGKKNKLKGYDYLD 360
Toxin_G         YDGLTGGVLSTSAYVEAGKGYIDKSGLADVVLGLGLSTAAIRGSNTFGKKQS------VS 354
Toxin_Omicron   YDGLTGGVLSTSAYVEAGKGYIDKSGLADVVLGLGLSTAAIRGSMTFGKKHKLKGYDYLD 360

Toxin_D         ---GPYKQNIK-NRYPNEVQQGKIF--DYTLENGQVKIRDGIKEVDFIIDLQGNLKVGRG 405
Toxin_E         ------------SQLDYVTNTGRL-------DGTLYSSKDLVLLENYLKKRGIELKIGDK 392
Toxin_F         DLLGDLNNKVKIKKYDSAESVNKYWH-QQNYDQPPYTPKTPVQDLELLIETKFVRVYDGG 419
Toxin_G         NVPNSSKNSAQYQKYKNELMKGDIL--E---NSKPIILGSDLKDSKVVSALTKNGSSMSD 409
Toxin_Omicron   DQLGSMKSHVKVNKYESSENANNWWKVEKGYDNPPYTSKTVVQDIRLLSDETFVRVYDGD 420

Toxin_D         HAHLSNGGDVQAA-GKLKVDSNGNVRRITNESGHYTP--TLGQAKNYQ-----QIFENTG 457
Toxin_E         YLPANKGGAFNAETGTIILKDNPTRYEVLHEVSHYIQYKNIGKGAYKNLPRAGKTFDTKQ 452
Toxin_F         SSKLHGGWLMKAEDIKGLTPTQIKDKFALPNMPKFVGEVTLPKGSNIRMGEVNPLFENKG 479
Toxin_G         WAKMESTYSYETSMGRGKIHFYKNLK--TGEINFYDVKMKVPIPKDLK-------IRNQ- 459
Toxin_Omicron   VSGLKGGWLMRAEDIRGLTPKQIQAKFALPAEPIYIGEVSLPKGSKLRIGEVAKNFGHKG 480

Toxin_D         ----INTKNAWLETYQLDVTKSG--YVDLAKL----KRIDSVKLK 492
Toxin_E         ----FNAPEQFVYDMLSNNTRRWKSFTEAERMHANWYINNFGGIR 493
Toxin_F         GGIQFDLKGQFIGE-----------FKELG------KISEWGGLK 507
Toxin_G         -----LTDDFWIVDLDNN-------FIPKG-------------VR 479
Toxin_Omicron   GGIQFDLKGQYIGD-----------YKEVG------KIIEWGK-- 506
```

B

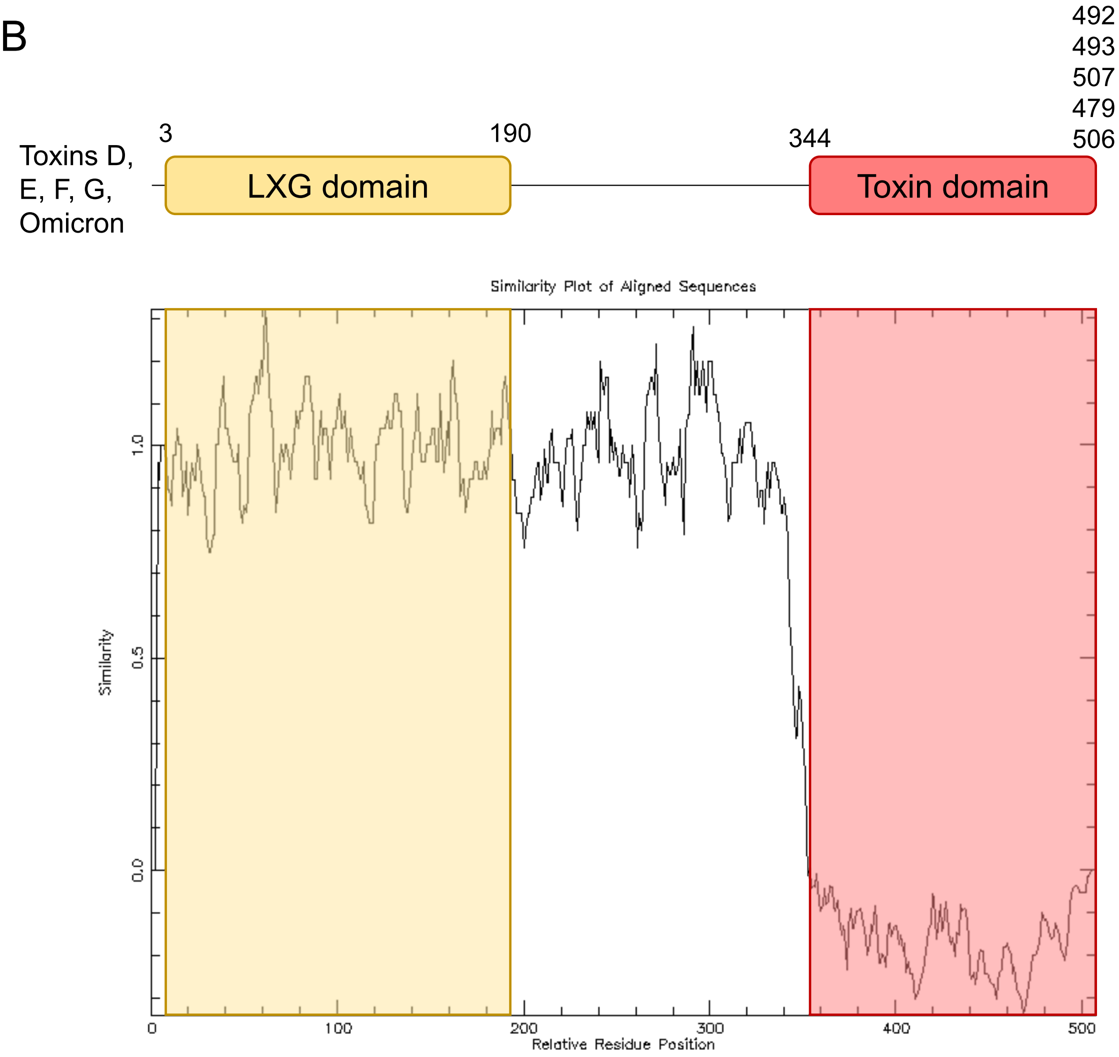

Figure S4. LXG toxins D, E, F, G and Omicron share a common N-terminal sequence. A. Sequence alignment of the five toxins. B. Analysis of the same five toxins using plotcon. The predicted boundary of the LXG domain is indicated.

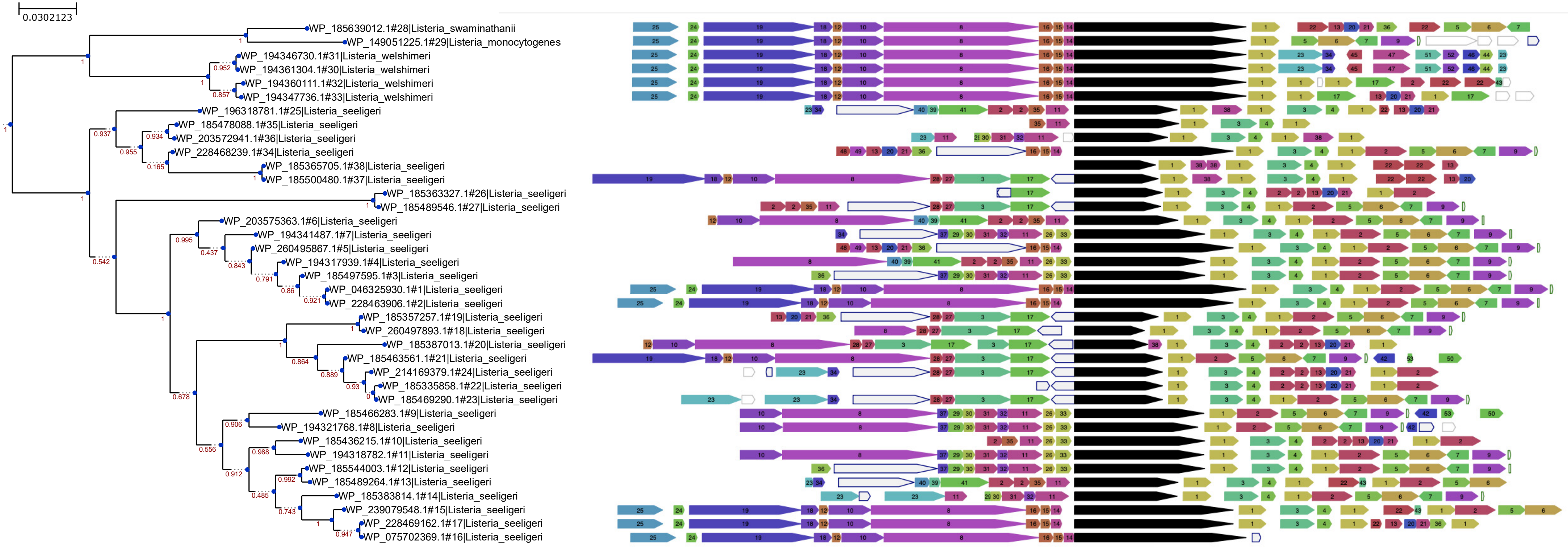

Figure S5. Genomic organisation of *lrhA* genes in non-monocytogenes species of *Listeria*. WebFlags was used to generate a genetic neighbourhood analysis of all LrhA homologues found in non-monocytogenes species. The tree was generated by webFlaGs using the ETE3 toolkit.

1(1) WP_203577659.1 hypothetical protein
1(1) WP_228463713.1 hypothetical protein
1(1) WP_185436222.1 hypothetical protein
1(1) WP_196318785.1 hypothetical protein
1(1) WP_185489543.1 hypothetical protein
1(1) WP_185469288.1 hypothetical protein
1(1) WP_194341489.1 hypothetical protein
1(1) WP_185415325.1 hypothetical protein
1(2) WP_185363328.1 hypothetical protein
1(2) WP_185365696.1 hypothetical protein
1(3) WP_185383815.1 hypothetical protein
1(2) WP_221633580.1 hypothetical protein
1(2) WP_185357268.1 hypothetical protein
1(1) WP_196318784.1 hypothetical protein
1(2) WP_185544005.1 hypothetical protein
1(1) WP_185387006.1 hypothetical protein
1(6) WP_046325931.1 hypothetical protein
1(1) WP_221638402.1 hypothetical protein
1(1) WP_185310098.1 hypothetical protein
1(1) WP_203572943.1 hypothetical protein
1(1) WP_194347735.1 hypothetical protein
1(1) WP_185383817.1 hypothetical protein
1(2) WP_194358207.1 hypothetical protein
1(2) WP_185365702.1 hypothetical protein
1(2) WP_194346731.1 hypothetical protein
1(1) WP_185466281.1 hypothetical protein
1(3) WP_185335851.1 hypothetical protein
1(1) WP_194341491.1 hypothetical protein
1(1) WP_185489260.1 hypothetical protein
1(1) WP_185489262.1 hypothetical protein
1(1) WP_185478090.1 hypothetical protein
1(1) WP_196318782.1 hypothetical protein
1(1) WP_185639011.1 hypothetical protein
1(1) WP_239077241.1 hypothetical protein
1(1) WP_194347732.1 hypothetical protein
1(1) WP_194347734.1 hypothetical protein
1(3) WP_185415324.1 hypothetical protein
1(1) WP_228469160.1 hypothetical protein
1(1) WP_185436216.1 hypothetical protein
1(2) WP_185365691.1 hypothetical protein
1(1) WP_203577657.1 hypothetical protein
1(1) WP_203575366.1 hypothetical protein
1(4) WP_228463346.1 hypothetical protein
1(1) WP_194360113.1 hypothetical protein
1(1) WP_203575364.1 hypothetical protein
1(4) WP_046325932.1 hypothetical protein
1(1) WP_149051226.1 hypothetical protein
1(1) WP_194358203.1 hypothetical protein
1(1) WP_194360112.1 hypothetical protein
1(1) WP_194317940.1 hypothetical protein

2(1) WP_221637190.1 hypothetical protein
2(2) WP_250210513.1 hypothetical protein
2(2) WP_221637815.1 hypothetical protein
2(1) WP_243285277.1 hypothetical protein
2(1) WP_214169381.1 hypothetical protein
2(1) WP_185489266.1 hypothetical protein
2(1) WP_228472257.1 hypothetical protein
2(1) WP_260448395.1 hypothetical protein
2(1) WP_185455567.1 hypothetical protein
2(1) WP_228474727.1 hypothetical protein
2(1) WP_221639762.1 hypothetical protein
2(4) WP_003745060.1 hypothetical protein
2(3) WP_012984615.1 hypothetical protein
2(1) WP_228463232.1 hypothetical protein
2(3) WP_012984616.1 hypothetical protein
2(3) WP_221633573.1 hypothetical protein
2(1) WP_185387010.1 hypothetical protein
2(1) WP_221645729.1 hypothetical protein
2(1) WP_185436218.1 hypothetical protein
2(1) WP_185455569.1 hypothetical protein
2(1) WP_221770661.1 hypothetical protein
2(1) WP_221642425.1 hypothetical protein
2(1) WP_260444442.1 hypothetical protein
2(1) WP_185363330.1 hypothetical protein
2(2) WP_221770127.1 hypothetical protein
2(1) WP_221645600.1 hypothetical protein
2(1) WP_221638398.1 hypothetical protein
2(1) WP_185335855.1 hypothetical protein
2(1) WP_221635928.1 hypothetical protein
2(1) WP_221639041.1 hypothetical protein
2(1) WP_228463708.1 hypothetical protein
2(1) WP_239077615.1 hypothetical protein
2(1) WP_038408420.1 hypothetical protein
2(1) WP_228478965.1 hypothetical protein

3(1) WP_185340229.1 hypothetical protein
3(2) WP_185463563.1 T7SS effector LXG polymorphic toxin
3(1) WP_185436217.1 hypothetical protein
3(1) WP_239077240.1 hypothetical protein
3(1) WP_228478964.1 hypothetical protein
3(1) WP_203575365.1 hypothetical protein
3(1) WP_260448413.1 hypothetical protein
3(1) WP_260442961.1 hypothetical protein
3(1) WP_185387014.1 T7SS effector LXG polymorphic toxin
3(1) WP_185478089.1 hypothetical protein
3(1) WP_185365694.1 hypothetical protein
3(1) WP_185489263.1 hypothetical protein
3(1) WP_250210512.1 hypothetical protein
3(1) WP_239079539.1 hypothetical protein
3(1) WP_228463347.1 hypothetical protein
3(1) WP_228463449.1 hypothetical protein
3(4) WP_185357254.1 T7SS effector LXG polymorphic toxin
3(1) WP_250209931.1 hypothetical protein
3(1) WP_260444553.1 hypothetical protein
3(1) WP_260443318.1 hypothetical protein
3(2) WP_228469720.1 hypothetical protein
3(1) WP_003750480.1 hypothetical protein
3(1) WP_228465423.1 hypothetical protein
3(1) WP_260448424.1 hypothetical protein
3(1) WP_260447974.1 hypothetical protein
3(6) WP_228463507.1 hypothetical protein
3(1) WP_214169378.1 T7SS effector LXG polymorphic toxin

4(3) WP_185357265.1 hypothetical protein
4(12) WP_003745059.1 hypothetical protein
4(4) WP_003750481.1 hypothetical protein
4(5) WP_185335856.1 hypothetical protein
4(1) WP_185451814.1 hypothetical protein
4(1) WP_185365693.1 hypothetical protein
4(1) WP_203572942.1 hypothetical protein
4(1) WP_185489544.1 hypothetical protein

5(2) WP_185544006.1 isocitrate lyase/phosphoenolpyruvate mutase family protein
5(1) WP_203575398.1 isocitrate lyase/phosphoenolpyruvate mutase family protein
5(1) WP_185463375.1 isocitrate lyase/phosphoenolpyruvate mutase family protein
5(1) WP_185489284.1 isocitrate lyase/phosphoenolpyruvate mutase family protein
5(1) WP_185639005.1 isocitrate lyase/phosphoenolpyruvate mutase family protein
5(1) WP_185469333.1 isocitrate lyase/phosphoenolpyruvate mutase family protein
5(1) WP_185489551.1 isocitrate lyase/phosphoenolpyruvate mutase family protein
5(4) WP_046325933.1 isocitrate lyase/phosphoenolpyruvate mutase family protein
5(1) WP_203577660.1 isocitrate lyase/phosphoenolpyruvate mutase family protein
5(1) WP_149051227.1 isocitrate lyase/phosphoenolpyruvate mutase family protein
5(1) WP_194341498.1 isocitrate lyase/phosphoenolpyruvate mutase family protein
5(1) WP_185383818.1 isocitrate lyase/phosphoenolpyruvate mutase family protein
5(1) WP_139591395.1 isocitrate lyase/phosphoenolpyruvate mutase family protein
5(1) WP_185463620.1 isocitrate lyase/phosphoenolpyruvate mutase family protein
5(1) WP_185415376.1 isocitrate lyase/phosphoenolpyruvate mutase family protein
5(3) WP_185335869.1 isocitrate lyase/phosphoenolpyruvate mutase family protein

6(1) WP_203577661.1 trifunctional transcriptional activator/DNA repair protein Ada/methylated-DNA--
6(7) WP_003745063.1 trifunctional transcriptional activator/DNA repair protein Ada/methylated-DNA--
6(1) WP_194341492.1 trifunctional transcriptional activator/DNA repair protein Ada/methylated-DNA--
6(1) WP_185461316.1 trifunctional transcriptional activator/DNA repair protein Ada/methylated-DNA--
6(1) WP_185363332.1 trifunctional transcriptional activator/DNA repair protein Ada/methylated-DNA--
6(1) WP_185463559.1 trifunctional transcriptional activator/DNA repair protein Ada/methylated-DNA--

6(1) WP_221771473.1 trifunctional transcriptional activator/DNA repair protein Ada/methylated-DNA--
6(3) WP_185357272.1 trifunctional transcriptional activator/DNA repair protein Ada/methylated-DNA--
6(1) WP_185335850.1 trifunctional transcriptional activator/DNA repair protein Ada/methylated-DNA--
6(1) WP_149051228.1 trifunctional transcriptional activator/DNA repair protein Ada/methylated-DNA--
6(2) WP_185383819.1 trifunctional transcriptional activator/DNA repair protein Ada/methylated-DNA--
6(2) WP_185469976.1 trifunctional transcriptional activator/DNA repair protein Ada/methylated-DNA--

7(3) WP_185357275.1 pentapeptide repeat-containing protein
7(2) WP_185455618.1 pentapeptide repeat-containing protein
7(4) WP_046325947.1 pentapeptide repeat-containing protein
7(1) WP_194332042.1 pentapeptide repeat-containing protein
7(1) WP_185469332.1 pentapeptide repeat-containing protein
7(1) WP_185383872.1 pentapeptide repeat-containing protein
7(3) WP_185415377.1 pentapeptide repeat-containing protein
7(2) WP_185469980.1 pentapeptide repeat-containing protein
7(1) WP_185350592.1 pentapeptide repeat-containing protein
7(1) WP_149051229.1 pentapeptide repeat-containing protein
7(1) WP_185639003.1 pentapeptide repeat-containing protein

8(1) WP_194347737.1 type VII secretion protein EssC
8(1) WP_194360110.1 type VII secretion protein EssC
8(1) WP_194318783.1 type VII secretion protein EssC
8(2) WP_046325927.1 type VII secretion protein EssC
8(1) WP_185466285.1 type VII secretion protein EssC
8(1) WP_185436428.1 type VII secretion protein EssC
8(1) WP_185387015.1 type VII secretion protein EssC
8(1) WP_203575361.1 type VII secretion protein EssC
8(1) WP_194321762.1 type VII secretion protein EssC
8(1) WP_260497892.1 FtsK/SpoIIIE domain-containing protein, partial
8(1) WP_185639016.1 type VII secretion protein EssC
8(1) WP_203577655.1 type VII secretion protein EssC
8(1) WP_149051221.1 type VII secretion protein EssC
8(1) WP_185364860.1 type VII secretion protein EssC
8(2) WP_075702371.1 type VII secretion protein EssC
8(1) WP_194346729.1 type VII secretion protein EssC
8(1) WP_185463564.1 type VII secretion protein EssC
8(1) WP_194361305.1 type VII secretion protein EssC

9(1) WP_149051269.1 2-hydroxyacid dehydrogenase family protein
9(1) WP_185387003.1 2-hydroxyacid dehydrogenase family protein
9(3) WP_185357278.1 2-hydroxyacid dehydrogenase family protein
9(1) WP_185455580.1 hydroxyacid dehydrogenase
9(2) WP_185415326.1 2-hydroxyacid dehydrogenase family protein
9(1) WP_185463558.1 hydroxyacid dehydrogenase
9(1) WP_139590304.1 2-hydroxyacid dehydrogenase family protein
9(3) WP_046325934.1 2-hydroxyacid dehydrogenase family protein
9(1) WP_185383820.1 hydroxyacid dehydrogenase
9(1) WP_185469287.1 hydroxyacid dehydrogenase
9(1) WP_075703054.1 hydroxyacid dehydrogenase
9(2) WP_185469977.1 hydroxyacid dehydrogenase
9(1) 2-hydroxyacid dehydrogenase family protein

10(1) WP_194321760.1 type VII secretion protein EssB
10(2) WP_075702372.1 type VII secretion protein EssB
10(1) WP_185433816.1 type VII secretion protein EssB
10(1) WP_139590297.1 type VII secretion protein EssB
10(1) WP_194318784.1 type VII secretion protein EssB
10(1) WP_012582211.1 type VII secretion protein EssB
10(1) WP_185307483.1 type VII secretion protein EssB
10(1) WP_185463565.1 type VII secretion protein EssB
10(1) WP_203575360.1 type VII secretion protein EssB
10(1) WP_185639017.1: type VII secretion protein EssB
10(1) WP_194360109.1 type VII secretion protein EssB
10(1) WP_011700924.1 type VII secretion protein EssB
10(2) WP_046325926.1 type VII secretion protein EssB
10(1) WP_185363042.1 type VII secretion protein EssB
10(2) WP_185337267.1 type VII secretion protein EssB

11(3) WP_185478087.1 hypothetical protein
11(3) WP_185365747.1 hypothetical protein
11(2) WP_185383809.1 hypothetical protein
11(1) WP_185383813.1 hypothetical protein
11(1) WP_194341483.1 hypothetical protein
11(1) WP_203572939.1 hypothetical protein
11(1) WP_185489265.1 hypothetical protein
11(2) WP_185436213.1 hypothetical protein
11(1) WP_185455574.1 hypothetical protein
11(2) WP_185497592.1 hypothetical protein

12(2) WP_003759563.1 EsaB/YukD family protein
12(1) WP_194347738.1 EsaB/YukD family protein
12(9) WP_003745045.1 EsaB/YukD family protein
12(1) WP_185442773.1 EsaB/YukD family protein
12(2) WP_003728973.1 EsaB/YukD family protein

13(1) WP_185363331.1 hypothetical protein
13(1) WP_185630933.1 hypothetical protein
13(1) WP_185337274.1 hypothetical protein
13(2) WP_185335854.1 hypothetical protein
13(2) WP_185415320.1 hypothetical protein
13(1) WP_196318786.1 hypothetical protein
13(2) WP_185365686.1 hypothetical protein
13(1) WP_185436219.1 hypothetical protein
13(1) WP_185387009.1 hypothetical protein
13(1) WP_185639009.1 hypothetical protein
13(1) WP_194358205.1 hypothetical protein

14(1) WP_185639013.1 hypothetical protein
14(7) WP_046325929.1 hypothetical protein
14(1) WP_149051224.1 hypothetical protein
14(4) WP_185331649.1 hypothetical protein

15(1) WP_185325795.1 hypothetical protein
15(5) WP_075702370.1 hypothetical protein
15(1) WP_185639014.1 hypothetical protein
15(2) WP_003745050.1 hypothetical protein
15(3) WP_185331648.1 hypothetical protein
15(1) WP_149051223.1 hypothetical protein

16(1) WP_149051222.1 hypothetical protein
16(4) WP_185325794.1 hypothetical protein
16(1) WP_185639015.1 hypothetical protein
16(7) WP_046325928.1 hypothetical protein

17(2) WP_185463562.1 hypothetical protein
17(1) WP_185335969.1 hypothetical protein
17(1) WP_194347731.1 hypothetical protein
17(1) WP_194347733.1 hypothetical protein
17(1) WP_194360114.1 hypothetical protein
17(7) WP_185340230.1 hypothetical protein

18(1) WP_185463566.1 type VII secretion protein EssA
18(1) WP_185639018.1 type VII secretion protein EssA
18(1) WP_194361306.1 type VII secretion protein EssA
18(2) WP_046325925.1 type VII secretion protein EssA
18(1) WP_070214792.1 type VII secretion protein EssA
18(3) WP_003750475.1 type VII secretion protein EssA
18(3) WP_011700923.1 type VII secretion protein EssA
18(1) WP_185337266.1 type VII secretion protein EssA

19(2) WP_046325924.1 type VII secretion protein EsaA
19(1) WP_203577654.1 type VII secretion protein EsaA
19(1) WP_185639019.1 type VII secretion protein EsaA
19(1) WP_185463567.1 type VII secretion protein EsaA
19(1) WP_194360108.1 type VII secretion protein EsaA
19(2) WP_075702373.1 type VII secretion protein EsaA
19(1) WP_185309473.1 type VII secretion protein EsaA
19(1) WP_149051220.1 type VII secretion protein EsaA
19(1) WP_185364862.1 type VII secretion protein EsaA
19(1) WP_185327606.1 type VII secretion protein EsaA
19(1) WP_194346728.1 type VII secretion protein EsaA

20(1) WP_185304653.1 hypothetical protein
20(1) WP_185387008.1 hypothetical protein
20(1) WP_194358204.1 hypothetical protein
20(2) WP_185415321.1 hypothetical protein
20(1) WP_185436220.1 hypothetical protein
20(1) WP_185468865.1 hypothetical protein
20(1) WP_185365684.1 hypothetical protein
20(1) WP_185639008.1 hypothetical protein
20(2) WP_185335853.1 hypothetical protein
20(1) WP_185337275.1 hypothetical protein
20(1) WP_214169382.1 hypothetical protein

21(3) WP_185337276.1 DUF4064 domain-containing protein
21(2) WP_185335852.1 DUF4064 domain-containing protein
21(1) WP_185432669.1 DUF4064 domain-containing protein
21(1) WP_185387007.1 DUF4064 domain-containing protein
21(1) WP_196318787.1 DUF4064 domain-containing protein
21(1) WP_214169383.1 DUF4064 domain-containing protein
21(1) WP_185436221.1 DUF4064 domain-containing protein
21(1) WP_185639007.1 DUF4064 domain-containing protein
21(1) WP_185314064.1 hypothetical protein

22(2) WP_185365688.1 DUF1672 family protein
22(1) WP_185639010.1 DUF1672 family protein
22(1) WP_185489261.1 DUF1672 family protein
22(2) WP_243285278.1 hypothetical protein
22(1) WP_203577658.1 DUF1672 family protein
22(2) WP_185365689.1 DUF1672 family protein
22(1) WP_194360115.1 DUF1672 family protein
22(1) WP_185451612.1 DUF1672 family protein
22(1) WP_185639006.1 DUF1672 family protein

23(2) WP_228469892.1 glycohydrolase toxin TNT-related protein
23(2) WP_228469894.1 glycohydrolase toxin TNT-related protein
23(1) WP_203572938.1 glycohydrolase toxin TNT-related protein, partial
23(1) WP_185383808.1 glycohydrolase toxin TNT-related protein
23(1) WP_214169400.1 glycohydrolase toxin TNT-related protein
23(2) WP_185489269.1 hypothetical protein
23(1) WP_185469291.1 glycohydrolase toxin TNT-related protein
23(1) WP_185469293.1 pre-toxin TG domain-containing protein
23(1) WP_260443317.1 TNT domain-containing protein, partial

24(1) WP_185639020.1 WXG100 family type VII secretion target
24(1) WP_003728976.1 WXG100 family type VII secretion target
24(4) WP_011700921.1 WXG100 family type VII secretion target
24(3) WP_003745040.1 WXG100 family type VII secretion target
24(2) WP_012984597.1 WXG100 family type VII secretion target

25(1) WP_185639021.1 adenylosuccinate synthase
25(2) WP_011700920.1 adenylosuccinate synthase
25(1) WP_194361307.1 adenylosuccinate synthase
25(1) WP_185331645.1 adenylosuccinate synthase
25(1) WP_003728977.1 adenylosuccinate synthase
25(5) WP_003745033.1 adenylosuccinate synthase

26(5) WP_012984619.1 hypothetical protein
26(3) WP_185335964.1 hypothetical protein

27(8) WP_185336754.1 hypothetical protein

28(8) WP_185336755.1 hypothetical protein

29(3) WP_185365739.1 hypothetical protein
29(1) WP_194341478.1 hypothetical protein
29(2) WP_185383878.1 hypothetical protein
29(2) WP_185497587.1 hypothetical protein

30(1) WP_194341479.1 hypothetical protein
30(3) WP_185365741.1 hypothetical protein
30(3) WP_185497588.1 hypothetical protein
30(1) WP_185383810.1 hypothetical protein

31(2) WP_185383811.1 hypothetical protein
31(1) WP_194341480.1 hypothetical protein
31(3) WP_185365743.1 hypothetical protein
31(2) WP_185497590.1 hypothetical protein

32(4) WP_185383812.1 toxin B
32(3) WP_185365745.1 toxin B
32(1) WP_194341481.1 toxin B

33(1) WP_194321766.1 hypothetical protein
33(2) WP_185497593.1 hypothetical protein
33(1) WP_194341485.1 hypothetical protein
33(2) WP_185365749.1 hypothetical protein
33(2) WP_185436214.1 hypothetical protein

34(5) WP_185336756.1 Imm59 family immunity protein
34(2) WP_185326090.1 Imm59 family immunity protein

35(5) WP_012984617.1 DUF5085 family protein
35(1) WP_185455572.1 DUF5085 family protein
35(1) WP_203575362.1 DUF5085 family protein

36(1) WP_185606003.1 hypothetical protein
36(1) WP_185337277.1 hypothetical protein
36(2) WP_185415322.1 hypothetical protein
36(1) WP_228469158.1 hypothetical protein
36(2) WP_185432670.1 hypothetical protein

37(1) WP_194341477.1 DUF4176 domain-containing protein
37(2) WP_185365737.1 DUF4176 domain-containing protein
37(1) WP_194321764.1 DUF4176 domain-containing protein
37(2) WP_185497585.1 DUF4176 domain-containing protein

38(1) WP_185500481.1 hypothetical protein
38(2) WP_196318783.1 hypothetical protein
38(1) WP_185365700.1 hypothetical protein
38(1) WP_185387012.1 hypothetical protein
38(1) WP_185365698.1 hypothetical protein

39(4) WP_012984613.1 hypothetical protein

40(3) WP_012984612.1 DUF5082 family protein
40(1) WP_185489268.1 DUF5082 family protein

41(2) WP_012984614.1 T7SS effector LXG polymorphic toxin
41(1) WP_196318779.1 T7SS effector LXG polymorphic toxin
41(1) WP_185489267.1 T7SS effector LXG polymorphic toxin

42(1) WP_185365828.1 site-specific integrase
42(1) WP_228463714.1 tyrosine-type recombinase/integrase
42(1) WP_185455581.1 site-specific integrase

43(1) WP_228474721.1 hypothetical protein
43(1) WP_203577694.1 hypothetical protein
43(1) WP_185489285.1 hypothetical protein

44(2) WP_185313123.1 hypothetical protein

45(2) WP_185326091.1 helix-turn-helix transcriptional regulator

46(2) WP_185327600.1 hypothetical protein

47(2) WP_185327601.1 replication initiation factor domain-containing protein

48(2) WP_185415318.1 DUF5082 family protein

49(1) WP_185434086.1 hypothetical protein
49(1) WP_185415319.1 hypothetical protein

50(1) WP_185463557.1 CPBP family intramembrane metalloprotease
50(1) WP_185455583.1 CPBP family intramembrane metalloprotease

51(2) WP_228469893.1 IS110 family transposase

52(2) WP_228469905.1 transposase

53(1) WP_260445213.1 hypothetical protein
53(1) WP_185365826.1 hypothetical protein

Fig S5.Text output.

A

```
essC8    1 MTRLQQNKVFQIEALQELLQTTKDLMNVAIFISENMDNEVRDLSNNIAEIPAEAKNHSLD
essC2      ------------------------------------------------------------
essC5      ------------------------------------------------------------
essC7      ------------------------------------------------------------

essC8   61 ALLDNKFEKSDFQDCINKLEKVLTKLIDEIPSYDKASGSLVKEITSITQRLNNTARQYKS
essC2      ------------------------------------------------------------
essC5      ------------------------------------------------------------
essC7      ------------------------------------------------------------

essC8  121 TIKTGMADISLDAYLTQLDNIRLATGADKTGKELDTILAGLKGLRVFSAEYSSDPVNLAT
essC2      ------------------------------------------------------------
essC5      ------------------------------------------------------------
essC7      ------------------------------------------------------------

essC8  181 GNFAYEKTDLVVGGATSLKLVRSYNSLNDQIGTLGKDWIHNFEMKFKILDDRIKVTFEDG
essC2      ------------------------------------------------------------
essC5      ------------------------------------------------------------
essC7      ------------------------------------------------------------

essC8  241 KVEEYIKDEEIYVAKKIDSGTIEKKDNGYILTCQDGKIYTFDLSGKWTEKEDRAGNRFVL
essC2      ------------------------------------------------------------
essC5      ------------------------------------------------------------
essC7      ------------------------------------------------------------

essC8  301 SYDKNGYLEKVRNREVAFYFSYDESRLVLVKDNSGREVKYSYEEGKLVKVENDLGQSYQF
essC2      ------------------------------------------------------------
essC5      ------------------------------------------------------------
essC7      ------------------------------------------------------------

essC8  361 EYDQFNKLKEIINPRDTKSVQNIFDSEGRTIKQFFPDGGEMSYEYVDEEQKIILTEQNGN
essC2    1 ----------------------------------------MSYEYVDEEQKIILTEQNGN
essC5      ------------------------------------------------------------
essC7      ------------------------------------------------------------

essC8  421 KIEYIHDDAFRNTEIIYEDGKIVRSFNEQGDKVKEIDKNGNVTEYNYDESGNITTIIRPI
essC2   21 KIEYIHDDAFRNTEIIYEDGKIVRSFNEQGDKVKEIDKNGNVTEYNYDESGNITTIIRPI
essC5      ------------------------------------------------------------
essC7      ------------------------------------------------------------

essC8  481 GEDVEIYYNDKNKITKVIENGEERTFLYNEKGRMISSKDGIGRVINIEYDSKGKIAKYTK
essC2   81 GEDVEIYYNDKNKITKVIENGEERTFLYNEKGRMISSKDGIGRVINIEYDSKGKIAKYTK
essC5      ------------------------------------------------------------
essC7      ------------------------------------------------------------

essC8  541 ADGSELEFTRDRKGNVTCLKNETGAKTEYEYDKLNRVIRSVDGNGNETKFTYDTSNRIVQ
essC2  141 ADGSELEFTRDRKGNVTCLKNETGAKTEYEYDKLNRVIRSVDGNGNETKFTYDTSNRIVQ
essC5      ------------------------------------------------------------
essC7      ------------------------------------------------------------

essC8  601 VENASKNTRKYTYNLADKITKIEDFNGEVISAEYNELNKISQLKNQAGDIVEYSYDLMWN
essC2  201 VENASKNTRKYTYNLADKITKIEDFNGEVISAEYNELNKISQLKNQAGDIVEYSYDLMWN
essC5      ------------------------------------------------------------
essC7      ------------------------------------------------------------
```

```
essC8  661 ISKIMDENGAEVKYLYDKLNRLACIQNPLNQEQKYSYDPNGNIREITQNNGSKIQYTYDE
essC2  261 ISKIMDENGAEVKYLYDKLNRLACIQNPLNQEQKYSYDPNGNIREITQNNGSKIQYTYDE
essC5    1 ----MDENGAEVKYLYDKLNRLACIQNPLNQEQKYSYDPNGNIREITQNNGSKIQYTYDE
essC7    1 ---------MDINFL---------------------------------------------

essC8  721 MNRRTSIQDADGTVTKYKYSKHGEIAEIEDALGNTTQFKYDSVGQMIEKVDKLGHKTTMS
essC2  321 MNRRTSIQDADGAVTKYKYSKHGEIAEIEDALGNTTQFKYDSVGQMIEKVDKLGHKTTMS
essC5   57 MNRRTSIQDADGAVTKYKYSKHGEIAEIEDALGNTTQFKYDSVGQMIEKVDKLGHKTTMS
essC7    7 ------------------------------------PFVFVS--------------TLLV

essC8  781 YTKLGKIETITYANGSVVSYSYYPGGKLKSVCLPDGNTETYEYDLNGNLSRKMNSLGHSY
essC2  381 YTKLGKIETITYANGSVVSYSYYPGGKLKSVCLPDGNTETYEYDLNGNLSRKMNSLGHSY
essC5  117 YTKLGKIETITYANGSVVSYSYYPGGKLKSVCLPDGNTETYEYDLNGNLSRKMNSLGHSY
essC7   17 YIQ----------------------------------------------SRKTNSLGHSY

essC8  841 TYVYDELDQVIEIINPEGNAREFCYDVVGNVTKTEDENGNITHYYYSCVGNLIRVVDALG
essC2  441 TYVYDELDQVIEIINPEGNAREFCYDVVGNVTKTEDENGNITHYYYSCVGNLIRVVDALG
essC5  177 TYVYDELDQVIEIINPEGNARKFCYDVVGNVTKTEDENGNITHYYYSCVGNLIRVVDALG
essC7   31 TYVYDELDQVIEIINPEGNAREFCYDVVGNVTKTEDENGNITHYYYSCVGNLIRVVDALG

essC8  901 NESCYDYNKTSQLTRVEQYGLIDAELGAVEKQKEAAQVTLYEYDLLGRVTSITDSLGNKE
essC2  501 NESCYDYNKTGQLTRVEQYGLIDAELGAVEKQKEAAQVTLYEYDLLGRVTSVTDSLGNKE
essC5  237 NESCYDYNKTGQLTRVEQYGLIDAELGAVEKQKEAAQVTLYEYDLLGRVTSITDSLGNKE
essC7   91 NESCYDYNKTGQLTRVEQYGLIDAELGAVEKQKEAAQVTLYEYDLLGRVTSVTDSLGNKE

essC8  961 LLEYDEMGNVVKKEDKEGFTTAYEYSETGNLTHILYADKKEVAFSYNPLKQLIQIEDWLG
essC2  561 LLEYNEMGNVVKKEDKEGFTTAYEYSETGNLTHILYADKKEVAFSYNPLKQLIQIEDWLG
essC5  297 LLEYDEMGNVVKKEDKEGFTTAYEYSETGNLTHILYADKKEVAFSYNPLKQLIQIEDWLG
essC7  151 LLEYDEMGNVVKKEDKEGFTTAYEYSETGNLTHILYADKKEVAFSYNPLKQLIQIEDWLG

essC8 1021 TTTAKLDALGRPLEIKDFKEEEVRYTWGSMGERTQVSYPDGTAVDYTYNEAMKLISLAVN
essC2  621 TTTAKLDALGRPLEIKDFKEEEVRYTWGSMGERTQVSYPDGTAVDYTYNEAMKLISLAVN
essC5  357 TTTAKLDALGRPLEIKDFKEEEVRYTWGSMGERTQVSYPDGTAVDYTYNEAMKLISLAVN
essC7  211 TTTAKLDALGRPLEIKDFKEEEVRYTWGSMGERTQVSYPDGTAVDYT-------------

essC8 1081 GEEKASYHYDWLGRLTSKVLGNGIQTDYTYNAFHRYESLIHHDREGILDRYDYHYDAVQN
essC2  681 GEEKASYHYDSLGRLTSKVLGNGIQTDYTYNAFHRYESLIHHDREGILDRYDYHYDAVQN
essC5  417 GEEKASYHYDSLGRLTSKVLGNGIQTDYTYNAFHRYESLIHHDREGILDRYDYHYDAVQN
essC7  258 -----------------------------YNAFHRYESLIHHDREGILDRYDYHYDAVQN

essC8 1141 KIGITKARRDLEEDTGEFRYHYDELHRLTHVWKDDTSIANYEYDAFGNRITSTEKQQSKQ
essC2  741 KIGITKARRDLEEDTGEFRYHYDELHRLTHVWKDDTSIANYEYDAFGNRITSTEKQQSKQ
essC5  477 KIGITKARRDLEEDTGEFRYRYDELHRLTHVWKDDTSIANYEYDAFGNRITSTEKQQSKQ
essC7  289 KIGITKARRDLEEDTGEFRYRYDELHRLTHVWKDDTSIANYEYDAFGNRTTSKEKQQSKQ

essC8 1201 YHYNKNNQLIETITNDEVQAFKYDKRGNLLEKRVQEEVDSTYTFDARNHLAKITQAGMQS
essC2  801 YHYNKNNQLIETITNDEVQAFKYDKRGNLLEKRVQEEVDSTYTFDARNHLAKITQAGMQS
essC5  537 YHYNKNNQLIETITNDEVQAFKYDKRGNLLEKRVQEEVDSTYTFDARNQLAKITQAGMQS
essC7  349 YHYNKNNQLVETITNGEVQTFKYDKRGNLLEKRVQEEVDSTYTFDARNQLAKITQAGMQS

essC8 1261 QFVYNGLGSRVERQKYDIDKQQLLASEKMLNDYTKTDHHLLTSQTEEGHQNYFWDNETIL
essC2  861 QFVYNGLGSRVERQKYDIDKQQLLASEKMLNDYTKTDHHLLTSQTEEGHQNYFWDNETIL
essC5  597 QFIYNGLGSRVERQQYDIEKQQLLTSERMLNDYTKTNHQLLTSQTEEGHQNYFWDNETVL
```

```
essC7  409 QFIYNGLGSRVERQQYDLEKQQLLTSERMLNDYTKTDHHLLTSQTEEGYQNYFWDNETIL

essC8 1321 GAISAEKDVEQFYLDDQLGSPIRLIENGNVQATYGYDTFGSPLSQTNSTPPRFGFTGHQT
essC2  921 GAISAEKDVEQFYLDDQLGSPIRLIENGNVQATYGYDTFGSPLSQTNSTPPRFGFTGHQT
essC5  657 GAISAEKDVEQFYLDDQLGSPIRLIENGNVQATYGYDTFGSPLSQTNSTPPRFGFTGHQT
essC7  469 GDISAEKDVEQFYLDDQLGSPIRLIENGNVQATYGYDTFGSPVSQTSSTPPRFGFTGHQT

essC8 1381 EEVGNLYFAEKRYLDAEEGRFISEDTVKGSIYVPKTMHAYAYCFNQPVDFIDTDGAFPSV
essC2  981 EEVGNLYFAEKRYLDAEEGRFISEDTVKGSIYVPKTMHAYAYCFNQPVDFIDTDGAFPSV
essC5  717 EEVGNLYFAEKRYLDAEEGRFISEDTVKGSIYVPKTMHAYAYCFNQPVDFIDTDGAFPSV
essC7  529 EEVGNLYFAEKRYLDAEEGRFISEDTVKGSIYVPKTMHAYAYCFNQPVDFIDTDGAFPSV

essC8 1441 KDFQDGLSNVKDSVVNAWDSGVDTVKDFVDNNVVGKEQVLLDEKVDKTKHKISQHIGGDL
essC2 1041 KDFQDGLSNVKDSVVNAWDSGVDTVKDFWDNNVVGTDQILKDDKEGNIAHRITQHVGGDF
essC5  777 KDFQDGLSNVKDSVVNAWDSGVDTVKDFWDNNVVGTDQILKDDKEGNIAHRITQHVGGDF
essC7  589 KDFQDGLSNVKDSVVNAWDSGVDTVKDFVDNNVVGTDQILKDTKVGNTTHRITQHIGGDI

essC8 1501 IVFESKGKSEKGSWSINFPEISVPFSDA--SISPSINLNDMSISNSYNFKGKNGEATSFA
essC2 1101 IVYEENSETEKGSWSINIPGISIPFGDTTNSMNTSINLTDKSITTSYNSNGKNGETVSLG
essC5  837 IVYEENSETEKGSWSINIPGISIPFGDTTNSMNTSINLTDKSITTSYNSNGKNGETVSLG
essC7  649 FVHEENKKEAKESWSINIPGISIPFGDTTYSTNTSINLKDKSITTSYNLNGKNGETTSSA

essC8 1559 MTMDKKGFDGSIKSGVTSVTGLSDMYIYSDSDTDIPWEDVPVKEILEALGYTVVGSMAII
essC2 1161 YTLDTKGGSYSMGSGVTSETGLSDM--YTDTEDYFHWEDMPVKEILEALGYSVVGSLAII
essC5  897 YTLDTKGGSYSMGSGVTSVTGLSDMYIYSDTDTDIPWEDVPVKEILEALGYTVVGSMAII
essC7  709 FTLDTKGLSRSIGSGVTSETGLSDM--YTNTEDYFHWEDVPVKEILEALGYTVVGSLAII

essC8 1619 GLAMDDATGVLIADDVLIPVAAKEIMKSWVRLGAACAGAI
essC2 1219 GLAMDDATGVLVADDVFIPVAAKEIMKSWVRVGAACAGGI
essC5  957 GLAMDDATGVLVADDVFIPVAAKEIMKSWVRVGAACAGGI
essC7  767 GLAMDDATGVLAADDVFIPVAAKEITKSWVRVGAACTGT-
```

B

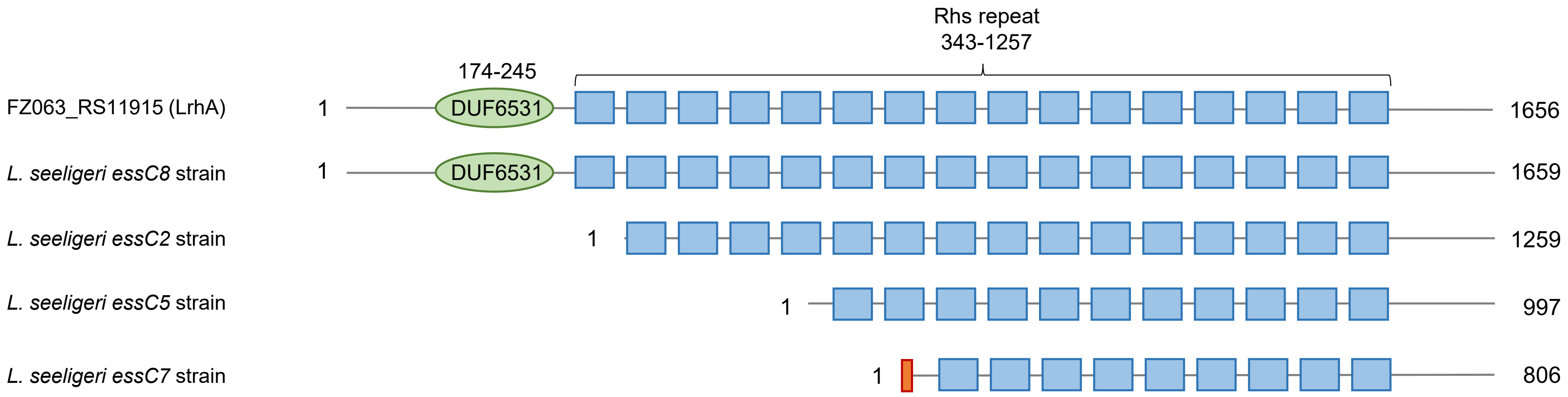

Figure S6. Truncated variants of LrhA are encoded at the *ess* locus in *L. seeligeri* genomes. A. A representative of each truncated LrhA from the different *essC* variants was aligned using MUSCLE v3.8.1551 and visualised using Boxshade. B. Schematic representation the truncated LrhA proteins encoded by *L. seeligeri essC2*, *essC5* and *essC7* in comparison to full length LrhA. The red box at the beginning of the *essC7* Rhs variant denotes a stretch of 19 amino acids which do not directly align to the consensus sequence.
